# Draft Genome Sequence of the Shellfish Bacterial Pathogen *Vibrio* sp. Strain B183

**DOI:** 10.1128/genomeA.00914-14

**Published:** 2014-09-18

**Authors:** Harold J. Schreier, Eric J. Schott

**Affiliations:** a Department of Marine Biotechnology, Institute of Marine and Environmental Technology, University of Maryland, Baltimore County, Baltimore, Maryland, USA; b Department of Biological Sciences, University of Maryland, Baltimore County, Baltimore, Maryland, USA; c University of Maryland Center for Environmental Science, Institute of Marine and Environmental Technology, Baltimore, Maryland, USA

## Abstract

We report the draft genome sequence of *Vibrio* sp. strain B183, a Gram-negative marine bacterium isolated from shellfish that causes mortality in larval mariculture. The availability of this genome sequence will facilitate the study of its virulence mechanisms and add to our knowledge of *Vibrio* sp. diversity and evolution.

## GENOME ANNOUNCEMENT

*Vibrio* sp. strain B183 is a marine bacterium isolated from diseased bay scallop (A*rgopecten irradians*) larvae and shown to cause mortality of oyster (*Crassostrea virginica*) larvae under mariculture conditions (1). Here we announce the genome sequence of strain B183 in order to facilitate identification of processes involved in pathogenesis and to add to our knowledge of *Vibrio* sp. diversity and evolution.

A single colony of strain B183 was grown in marine broth 2216 (Difco) at 28°C and DNA was extracted using the Wizard genomic DNA purification kit (Promega). Sequencing was done with an Illumina MiSeq benchtop sequencer. The read library comprised 5,580,583 (2 × 250-bp) fragments, representing one of the largest *Vibrio* sp. genomes to date, with average coverage of 840×. *De novo* assembly of the paired reads was done using the CLC Genomics Workbench assembly tool (CLC Bio/Qiagen), yielding 52 contigs with an average length of 107,309 bp. The *N*_50_ is 292,693 bp with a G+C composition of 45.2%. Gene prediction and annotation using the RAST (Rapid Annotation using Subsystem Technology) server (2) generated 5,143 protein encoding genes and 81 transfer and ribosomal RNA genes. The closest relative analyzed by the SEED viewer 2.0 program (3) was coral pathogen *Vibrio coralliilyticus* strain ATCC BAA-450 (score = 526).

The B183 genome carries quorum-sensing and biofilm production–associated genes including *luxU*, *luxO*, *luxT*, *luxN*, *luxR*, and *hapR* regulators (4), the gene for *N*-(3-hydroxybutanoyl)-L-homoserine lactone (autoinducer-1) synthase, genes for mannose-sensitive hemaglutinin (MSHA) biogenesis and pilin proteins (5), and gene clusters associated with capsule polysaccharide production (*cps*, *vps*, and *eps*) (6). The *syp* gene cluster for symbiotic colonization (7) was identified and genes for ABC and siderophore transporters, receptors, and the Fur regulator were found, which may also contribute to colonization (8).

While *Vibrio* CTX phage (9) and zona occludens toxin genes appear to be absent in the B183 genome, the RTX toxin was identified and the PHAST search tool (10) revealed an intact phage genome related to the *Vibrio cholerae* K139 lysogenic phage (11). Virulence-related secretory HlyD, at least seven hemolysins, the *toxRS* virulence regulator, and genes encoding types I, II, III, and VI secretion system components were found. Genes for proteases important for *Vibrio* pathogenicity (12) were identified, including metalloproteases, collagenases, and four vibriolysins, as well as ten chitinase-encoding genes, a virulence inventory that is comparable to that found for *V. coralliilyticus* (13).

The genome encodes 1,484 hypothetical proteins (from 113 to 4451 aa) with no significant similarity to any protein in GenBank (28.8% of the open reading frames [ORFs]). Studies focusing on these unknown ORFs as well as the investigation of specific pathways defined by the genes mentioned above will provide insight to their contribution to the pathogenicity of B183. Development of molecular tools to track and enumerate B183 in *in vivo* challenges of oysters and other hosts is being conducted to assist in these investigations.

### Nucleotide sequence accession numbers.

This whole-genome shotgun project has been deposited at DDBJ/EMBL/GenBank under the accession number JPQB00000000. The version described in this paper is the first version, JPQB01000000.
